# Aneuploidy promotes intraspecific diversification of the endemic East Asian herb *Lycoris aurea* complex

**DOI:** 10.3389/fpls.2022.955724

**Published:** 2022-09-29

**Authors:** Jinxia Wang, Lu Sun, Hao Zhu, Yanni Lv, Weiqi Meng, Guosheng Lv, Dong Zhang, Kun Liu

**Affiliations:** ^1^Anhui Provincial Key Laboratory of the Conservation and Exploitation of Biological Resources, College of Life Sciences, Anhui Normal University, Wuhu, China; ^2^Collaborative Innovation Center of Recovery and Reconstruction of Degraded Ecosystem in Wanjiang Basin Co-founded by Anhui Province and Ministry of Education, Anhui Normal University, Wuhu, China; ^3^Nanjing Institute of Environmental Sciences, Ministry of Ecology and Environment of the People’s Republic of China, Nanjing, China; ^4^Genomics and Genetic Engineering Laboratory of Ornamental Plants, Department of Horticulture, College of Agriculture and Biotechnology, Zhejiang University, Hangzhou, China

**Keywords:** *Lycoris aurea complex*, aneuploidy, cytogeography, cpDNA haplotype, leaf morphology, intraspecific differentiation

## Abstract

Polyploidy has received considerable interest in the past, but aneuploidy and partial rearrangements may also influence genomic divergence. In this study, we reported a comprehensive cytogeographic, morphological and genetic analysis of *Lycoris aurea* complex throughout its range and attempted to explore the association between aneuploidy and species diversification. The karyotypes of this complex presented aneuploidy variations mainly divided into four cytotypes: I (2*n* = 10m + 2T), II (2*n* = 8m + 6T), III (2*n* = 7m + 8T), and IV (2*n* = 6m + 10T). Cytotype distributions were highly structured geographically. Two main cytotypes, II and IV, are geographically allopatric. The populations with cytotype II are mainly distributed in central China and the southern islands of Japan. Cytotypes IV is disjunctly distributed in southwestern and southeastern China. The cytotypes with fewer chromosome numbers tend to occur at high latitudes. For analyzing the phylogeographic pattern and genetic structure of this complex, we sequenced four chloroplast DNA fragments (4,748 bp in total) of 241 individuals from 42 populations. Extremely high diversity of cpDNA haplotypes was found, with genetic diversity index (*H*_d_) being 0.932 and 98.61% of the genetic variation occurring among populations, indicating that this complex has undergone strong intraspecific differentiation. The cytotype II had the highest haplotype diversity (*H*_d_ = 0.885), while cytotype IV harbored the highest nucleotide diversity (π = 4.09 × 10^–3^). We detected significant leaf morphological differences not only between cytotype II and IV but also between west lineage and east lineage within cytotype IV. These results illustrated that aneuploidy contributed to extensive morphological and genetic differentiation in *L*. *aurea* complex. It was suggested that *L. aurea* complex should comprise multiple independent evolutionary lineages, and accurate species delimitation needs to be established further in an integrative taxonomic approach.

## Introduction

Variations of karyotype and chromosome number, such as polyploidy and aneuploidy, can drive genomic novelty and intraspecific diversification and act as a mode of immediate and sympatric species (Soltis et al., 2015; do Pico et al., 2019). Unrecognized cytotype variation can lead to an underestimation of species richness (Soltis et al., 2007, 2010). Hence, more cytogeographical studies of species or species complexes across their entire geographic range are needed to detect and understand patterns of cytotype formation, establishment, and migration (Wefferling et al., 2017). For several decades, researchers have paid more attention to the polyploid formation and elucidating the consequences of polyploidy, showing that polyploidization may be accompanied by a shift in morphology, phenology and ecology (Soltis et al., 2010; Thompson et al., 2014; Wefferling et al., 2017). Compared to well-studied polyploidy, aneuploidy variation and its long-term consequences are less investigated in plants, resulting in huge gaps in our knowledge about the association between aneuploidy and species diversification.

*Lycoris aurea* (L’Hér.) Herb., a member of the family Amaryllidaceae, is a typical aneuploid complex with the chromosome numbers of 2*n* = 12–16, including *L*. *aurea* var. *aurea*, *L*. *aurea* var. *angustitepala* and *L*. *traubii* (Kurita, 1987; Hsu et al., 1994; Ji and Meerow, 2000). Differing from *L*. *aurea*, the leaves of *L*. *traubii* appear in autumn, about a month later than in *L*. *aurea* and there is no remains of leaf-bases in *L*. *traubii* (Hsu et al., 1994). The *L*. *aurea* complex, also called “Golden Spider Lily,” is distributed from southwestern China eastward to the northern tip of Taiwan and the southern islands of Japan (Kurita, 1987; Hsu et al., 1994). *L*. *aurea* is an important groundcover and ornamental flower plant, widely applied in many fields, including landscape garden, industry and agriculture (Quan and Liang, 2017). Except for horticultural values of importance, *L*. *aurea*, as a medicinal species of the Amaryllidaceae family, is used in the practice of traditional Chinese medicine (TCM) because of its broad pharmacological activities of Amaryllidaceae alkaloids (Wang et al., 2017). Lycorine and Galantamine, which are rich in *Lycoris* bulbs, have been reported to exhibit immunostimulatory, antimalarial, tumor, and viral activities. For example, galanthamine, a cholinesterase inhibitor, has been clinically used in the treatment of Dutch patients with mild to moderate Alzheimer’s disease (Wang et al., 2017). Inariyama was the first cytologist who counted the somatic chromosome number of 12 in this taxon (Kurita, 1987). Cytogeographic patterns in the southern islands of Japan have been depicted clearly, based on 5 populations, and three cytotypes were discovered (Kurita, 1987). The chromosome numbers of this complex have been reported sporadically (Kurita, 1987; Hsu et al., 1994; Shu, 2010), but no previous studies have addressed the cytogeography of the complex.

The current study examined the cytotype diversity and distribution of *L. aurea* complex and tested how aneuploidy variation correlated with and may have affected the population genetic structure and the leaf morphology of the complex. We specifically addressed the following questions. (1) How many different cytotypes and what cytogeographic patterns are present across the entire distribution range of the complex? (2) Is the cytogeographic pattern concordant with the population genetic structure revealed in cpDNA variation? (3) Are the different cytotypes recognizable using morphological characters? To achieve these goals, we undertook a large-scale screening covering most of the distribution range of the *L*. *aurea* aneuploid complex, assessing the association between aneuploidy and intraspecific diversification.

## Materials and methods

### Plant materials

Forty-six natural populations of *L*. *aurea* complex through its distribution range in East Asia, including forty-five populations in China and one population in Japan were collected. To avoid biasing sampling, bulbs were collected apart from more than five meters each other and then transplanted in an experimental common-garden in Anhui Normal University. Detailed information for populations sampled in this study is listed in Table 1.

**TABLE 1:** Origins of materials and the karyotypes.

Population	Localities	Longitude (°)	Latitude (°)	Individuals analyzed	Karyotypes	Figures
P1	Laizhou Town, Nanping, Fujian, China	118.13	26.68	6	2*n* = 15 = 7m + 8T	Figure 1A
P2	Luyuan Village, Shaoguan, Guangdong	114.10	25.10	6	2*n* = 15 = 7m + 8T	Figure 1B
P3	Lvtian Town, Conghua, Guangdong, China	113.92	23.80	6	2*n* = 16 = 6m + 10T	Figure 1C
P4	Danxiashan, Guangdong, China	113.73	25.02	6	2*n* = 16 = 6m + 10T	Figure 1D
P5	Huashan Town, Shaoguan, Guangdong, China	113.99	24.92	6	2*n* = 16 = 6m + 10T	Figure 1E
P6	Yueli Town, Baise, Guangxi, China	106.24	24.28	6	2*n* = 14 = 8m + 6T	Figure 1F
P7	Maocaoping Village, Baise, Guangxi, China	105.63	24.50	6	2*n* = 14 = 8m + 6T	Figure 1G
P8	Tongxiang Village, Baise, Guangxi, China	105.67	24.52	6	2*n* = 15 = 7m + 8T	Figure 1H
P9	Wutong Town, Guilin, Guangxi, China	110.07	25.37	6	2*n* = 14 = 8m + 6T	Figure 1I
P10	Rongjiang Town, Guilin, Guangxi, China	110.32	25.68	3	2*n* = 14 = 8m + 6T	Figure 1J
P11	Tongde Town, Jingxi County, Guangxi, China	106.59	23.08	6	2*n* = 15 = 7m + 8T	Figure 1K
P12	Rongan County, Liuzhou, Guangxi, China	109.40	25.22	6	2*n* = 14 = 8m + 6T	Figure 1L
P13	Qingrengu, Guiyang, Guizhou, China	106.81	26.60	6	2*n* = 16 = 6m + 10T	Figure 1M
P14	Pan County, Liupanshui, Guizhou, China	104.67	25.78	3	2*n* = 16 = 6m + 10T	Figure 1N
P15	Pingxi Village, Qindongnan, Guizhou, China	107.80	27.13	6	2*n* = 14 = 8m + 6T	Figure 1O
P16	Boyang Town, Qinxinan, Guizhou, China	105.36	25.67	6	2*n* = 16 = 6m + 10T	Figure 1P
P17	Zhaibao Village, Tongren, Guizhou, China	108.75	27.77	6	2*n* = 14 = 8m + 6T	Figure 1Q
P18	Wufeng County, Yichang, Hubei, China	110.67	30.20	6	2*n* = 14 = 8m + 6T	Figure 1R
P19	Wudangshan, Shiyan, Hubei, China	111.04	32.48	6	2*n* = 14 = 8m + 6T	Figure 1S
P20	Xingshan County, Yichang, Hubei, China	110.88	31.23	6	2*n* = 14 = 8m + 6T	Figure 1T
P21	Shadaogou Town, Enshi, Hubei, China	109.61	29.68	6	2*n* = 14 = 8m + 6T	Figure 2A
P22	Xiaoping Village, Yichang, Hubei, China	111.75	31.17	6	2*n* = 14 = 8m + 6T	Figure 2B
P23	Maogou Town, Xiangxi, Hunan, China	109.38	28.58	6	2*n* = 14 = 8m + 6T	Figure 2C
P24	Cili County, Changde, Hunan, China	111.25	29.53	6	2*n* = 14 = 8m + 6T	Figure 2D
P25	Dankou Town, Shaoyang, Hunan, China	110.24	26.33	6	2*n* = 14 = 8m + 6T	Figure 2E
P26	Dao County, Yongzhou, Hunan, China	111.56	25.50	6	2*n* = 14 = 8m + 6T	Figure 2F
P27	Jinbaotang Town, Yongzhou, Hunan, China	112.10	26.42	6	2*n* = 12 = 10m + 2T	Figure 2G
P28	Xinhuang County, Huaihua, Hunan, China	109.22	27.27	6	2*n* = 14 = 8m + 6T	Figure 2H
P29	Bozhou Town, Huaihua, Hunan, China	109.17	27.37	6	2*n* = 14 = 8m + 6T	Figure 2I
P30	Longtan Village, Zhuzhou, Hunan, China	113.77	26.20	6	2*n* = 14 = 8m + 6T	Figure 2J
P31	Jiemuxi, Yuanling County, Hunan, China	110.45	28.85	6	2*n* = 14 = 8m + 6T	Figure 2K
P32	Daping Town, Zhangjiajie, Hunan, China	110.52	29.00	6	2*n* = 14 = 8m + 6T	Figure 2L
P33	Jinbianxi, Zhangjiajie, Hunan, China	110.49	29.35	6	2*n* = 14 = 8m + 6T	Figure 2M
P34	Wulingyuan, Zhangjiajie, Hunan, China	110.42	29.38	6	2*n* = 14 = 8m + 6T	Figure 2N
P35	Dongxi Town, Guangyuan, Sichuan, China	106.25	32.05	6	2*n* = 14 = 8m + 6T	Figure 2O
P36	Motan Town, Guangyuan, Sichuan, China	106.05	32.17	6	2*n* = 14 = 8m + 6T	Figure 2P
P37	Nanchong, Sichuan, China	106.05	30.80	6	2*n* = 14 = 8m + 6T	Figure 2Q
P38	Qingchengshan, Dujiangyan, Sichuan, China	103.56	30.87	6	2*n* = 14 = 8m + 6T	Figure 2R
P39	Huagaoxi, Xuyong County, Sichuan, China	105.54	28.27	6	2*n* = 16 = 6m + 10T	Figure 2S
P40	Pingbian Coungy, Yunnan, China	103.69	22.93	6	2*n* = 16 = 6m + 10T	Figure 2T
P41	Malipo Coungy, Wenshan, Yunnan, China	104.73	23.05	6	2*n* = 16 = 6m + 10T	Figure 2U
P42	Yuxi, Yunnan, China	102.45	24.27	6	2*n* = 16 = 6m + 10T	Figure 2V
P43	Maji Town, Fugong County, Yunnan	98.89	27.30	6	2*n* = 16 = 6m + 10T	Figure 2W
P44	Luoping County, Qujing, Yunnan	104.40	25.00	6	2*n* = 16 = 6m + 10T	Figure 2X
P45	Yinmu Village, Pengshui County, Chongqing, China	108.20	29.50	2	2*n* = 14 = 8m + 6T	Figure 2Y
P46	Kagoshima, Japan	130.46	31.23	4	2*n* = 14 = 8m + 6T	Figure 2Z
P47	Jinfoshan, Chongqing, China	107.11	29.05		2*n* = 14 = 8m + 6T	Shu, 2010
P48	Jiulianshan, Quannan County, Jiangxi, China	114.59	24.63		2*n* = 15 = 7m + 8T	Shu, 2010
P49	Emeishan, Sichuan, China	103.37	29.58		2*n* = 16 = 6m + 10T	Shu, 2010
P50	Ikenouchi, Kagoshima, Japan	130.44	31.25		2n = 12 = 10m + 2T; 2n = 13 = 9m + 4T	Kurita, 1987
P51	Yozadake, Okinawa island, Okinawa, Japan	127.70	26.13		2*n* = 14 = 8m + 6T	Kurita, 1987
P52	Omotodake, Ishigakijima, Okinawa, Japan	124.19	24.40		2*n* = 14 = 8m + 6T	Kurita, 1987
P53	Tonaki village, Tonaki island, Okinawa, Japan	127.15	26.36		2*n* = 14 = 8m + 6T	Kurita, 1987

P47–P53 from previously published data.

### Karyotype analysis

The karyotypes were determined at mitotic metaphase for a total of 264 bulbs from the 46 populations (2–6 bulbs for each population) using conventional karyotype analysis methods (Zhou et al., 2007; Liu et al., 2019). The karyotype formula was based on the measurements of mitosis metaphase chromosomes taken from more than three well-spread metaphase cells. For the karyotype description and comparison, the simplified symbols were adapted according to Levan et al. (1964) and Liu et al. (2012): m for large metacentric chromosome with arm ratio of 1.00–1.70; T for telocentric chromosome having mostly terminal centromere with a dot-like short arm whose length is very short and with the arm ratio being more than 20.0; B for very small chromosome; r means arm ratio.

### Analysis of cytotype distribution

The relationship between latitude and cytotypes was tested using Pearson Correlation Analysis by SPSS v17.0. To exactly reveal the geographical distribution patterns of each cytotype of *L. aurea* complex in East Asia, we chose 7 previously published populations with precise chromosome number data and geographical localities, of which 3 populations (Shu, 2010) and 4 populations (Kurita, 1987) were from China and Japan, respectively. In total, 53 populations with exact karyotypic data were mapped using ArcMap 10.0.

### DNA extraction, amplification and sequencing

After karyotypic analysis, we analyzed the plastid *matK*, *rpl32-trnL*, *psbA-trnH*, and *ndhF* DNA regions of 241 individuals from 42 out of 46 populations and one outgroup species, *Shoubiaonia yunnanensis* (Qin et al., 2021), in addition to plastome sequences of five other *Lycoris* species released by our lab recently (Liu et al., 2022). All samples of DNA were extracted from fresh leaf materials using the modified 2x CTAB method. All four DNA regions were amplified from total genomic DNA, using the following PCR and sequencing methods. (1) *matK* was amplified using primers *matK*-1F and *matK*-R, and sequenced with primers *matK*-1F, *matK*-2F and *matK*-R. (2) *rpl32-trnL* was amplified and sequenced with primers *rpl32*-F and *trnL*-R. (3) *psbA-trnH* was amplified and sequenced with primers *psbA*-F and *trnH*-R. (4) *ndhF* was amplified with primers *ndhF*-1F and *ndhF*-R, and the more than 2000 base-pair PCR products were sequenced with primers *ndhF*-1F, *ndhF*-2F, *ndhF*-3F, and *ndhF*-4F. The Primer sequences were listed in Supplementary Table 1. The amplification was performed using the polymerase chain reaction (PCR) in 20 μl reaction mixtures containing 10 μl DreamTaq Green PCR Master Mix (2X) (Thermo Fisher Scientific), 0.5 mM per primer, 1 ng DNA and water (nuclease-free). PCR was carried out under the following conditions: one cycle at 95°C for 2 min, 35 cycles of 95°C for 25 s, 58°C for 25 s, 72°C for 1 min, and a final cycle at 72°C for 3 min. Purified PCR products were sequenced using standard methods by Sangon Biotech.^1^ All sequences newly obtained have been deposited in GenBank. The accession numbers are OP021502–OP021631.

### Phylogeographical surveys

Sequences were aligned and adjusted using the Sequencher software, then assembled and checked manually, and the uncertain parts of both ends of these sequences were deleted. To assess degrees and patterns of diversity in the cpDNA data matrices of *L*. *aurea* complex, we calculated the number of haplotypes, haplotype diversity (*H*_d_) and nucleotide diversity (π) at species and cytotype levels using Dnasp v.5.1 (Rozas et al., 2003). Genealogical relationships of the haplotypes identified were inferred from a 95% statistical parsimony network constructed in TCS v.1.21 (Clement et al., 2000), with gaps (indels) excluded. To quantify the genetic differentiation partitioned among different groups and total genetic variance, analyses of molecular variance (AMOVA) were performed with ARLEQUIN (version 3.5) (Excoffier and Lischer, 2010), and the significance of variance components was tested with 10,000 permutations.

### Phylogenetic analyses

Phylogenetic relationships among the cpDNA haplotypes were reconstructed by Maximum Likelihood (ML) and Bayesian inference (BI) methods, respectively. The ML analyses were performed using RAxML-HPC v8.2.10 (Stamatakis, 2014) with 1000 bootstrap replicates at the CIPRES Science Gateway website (Miller et al., 2010), under the GTR + G substitution model determined by the Akaike Information Criterion (AIC) in jModelTest 2.1.7 (Darriba et al., 2012). Bayesian analysis (BI) was conducted using the NSF Extreme Science and Engineering Discovery Environment (XSEDE) application of MrBayes v.3.2.7a (Ronquist et al., 2012) provided by the CIPRES Science Gateway (Miller et al., 2010). Two independent Markov Chain Monte Carlo chains were calculated simultaneously for 1,000,000 generations and sampled every 100 generations. The first 25% of calculated trees were discarded as burn-in, and a consensus tree was constructed using the remaining trees.

### Leaf morphological and anatomical measurements

To measure the length and width of the leaves, randomly selected six leaves from 6 plants (one leaf per individual) within one population were measured using a metric ruler. For anatomical measurements, three leaf segments from the middle of leaves of three individuals for each population were fixed in FAA (70% ethanol:formaldehyde:acetic acid: glycerol, 90:5:5:5). The samples of leaves were embedded on the sample holder with OCT compound (SAKURA) as an embedding medium, and the temperature in the cryostat microtome (Leica CM1950) chamber was set to –20^°^C, and then sliced after the materials were solidified. The section thickness was set to be ∼18 μm, during specific operation, appropriate adjustments of the thickness were made according to the differences of leaves from different populations. Then the sections were stained with 5% safranin and made into tablets. The sections were observed with a computer-equipped Leica microscope (Leica DMi8). Stomatal and epidermal cell densities were estimated from abaxial leaf surface impressions of six leaf segments. The photographs were taken under a biological microscope (Olympus) at 100 × magnification for morphological measurements. Micrographs were analyzed with Olympus-affiliated software to determine stomatal and epidermal cell densities. Stomatal index was calculated by using the formula [S/(E + S)] × 100, where S and E indicate the number of stomata and epidermal cells per unit area of leaf, respectively.

## Results

### Cytotype distribution patterns in *Lycoris aurea* complex

**FIGURE 1:**
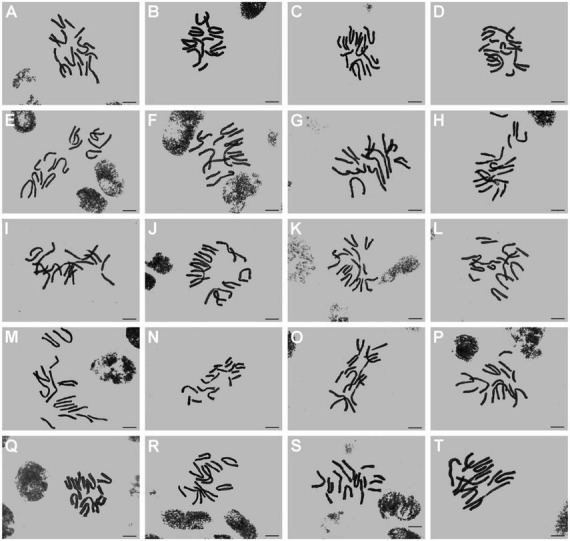
Somatic chromosomes of *L. aurea* complex. **(A–T)** From population 1 to population 20. Scale bar 10 μm.

**FIGURE 2:**
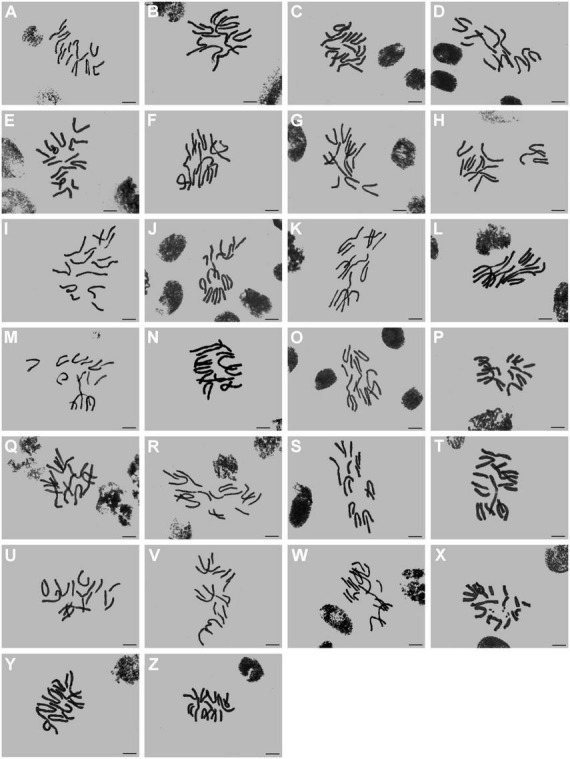
Somatic chromosomes of *L. aurea* complex. **(A–Z)** From population 21 to population 46. Asterisk indicates B chromosome in Figure 2X. Scale bar 10 μm.

**FIGURE 3:**
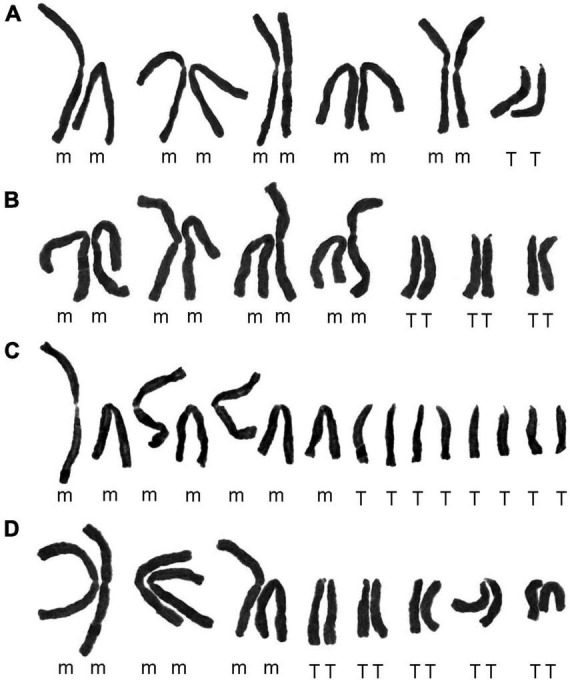
Karyograms of four representative cytotypes. **(A)** Cytotype I from population 27 (Figure 2G); **(B)** cytotype II from population 23 (Figure 2C); **(C)** cytotype III from population 11 (Figure 1K); **(D)** cytotype IV from population 4 (Figure 1D).

**FIGURE 4:**
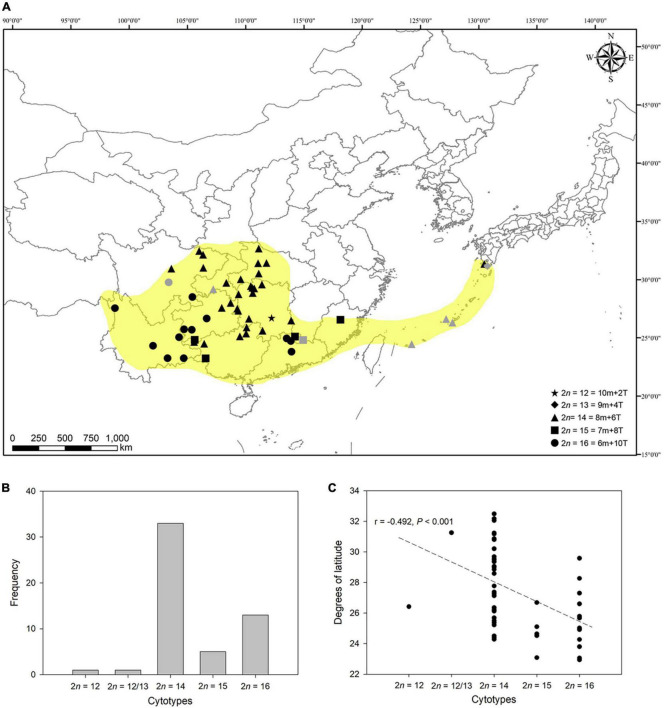
Cytogeographical pattern of *L. aurea* complex in East Asia. **(A)** Distribution map of cytologically investigated and previously published populations of this complex. Gray marks represent published karyological data. Yellow-shaded areas show the distribution range of this complex to our knowledge. The map image was generated by ArcGIS v.9.3 (http://www.esri.com/software/arcgis/arcgis-for-desktop). **(B)** Histogram of frequencies of cytotypes of 53 populations of *L. aurea* complex cytogeographically analyzed. **(C)** Scatter plot of cytotype (chromosome numbers) vs. latitude (degrees). The linear relationship shows a significantly negative association between the chromosome numbers and altitude (*r* = –0.492, *P* < 0.001).

In this study, the somatic chromosome numbers of 264 bulbs from 46 locations were reported (Figures 1, 2 and Table 1). The chromosome numbers of the examined bulbs ranged from 2*n* = 12 to 2*n* = 16. Of the 46 populations analyzed, four cytotypes of four different chromosome numbers were identified: (1) 2*n* = 12 = 10m + 2T (cytotype I; Figure 3A); (2) 2*n* = 14 = 8m + 6T (cytotype II; Figure 3B); (3) 2*n* = 15 = 7m + 8T (cytotype III; Figure 3C); (4) 2*n* = 16 = 6m + 10T (cytotype IV; Figure 3D), indicating that the karyotypic diversity of the complex is extremely high, presenting aneuploidy variation. The B-chromosomes were detected in one bulb from population 44 with cytotype IV (Figure 2X). All 46 localities in our study were characterized by a single cytotype, and no mixed-cytotype population was found. The measured and calculated values of each chromosome of the representative four cytotypes are summarized in Supplementary Table 2.

To exactly reveal the cytogeographical pattern of the complex, we analyzed 53 populations of this complex (see Table 1). As depicted in Figure 4A, despite the great diversity of cytotypes found, cytotype II (2n = 14) and cytotype IV (2n = 16) are the most frequent among populations (Figure 4B). Of all the populations of the complex analyzed cytogeographically (46 counts made in this study and 7 previously reported), 62.26% corresponded to populations with cytotype II, 24.53% were cytotype IV, 9.43% cytotype III, 1.89% cytotype I, and 1.89% corresponded to the mixed population (2n = 12/13).

The populations with cytotype II are mainly distributed in central regions of China and the southern islands of Japan. The populations with cytotype III and IV are disjunctly distributed in southwestern and southeastern China. From a limited region in southern Hunan, only one population having 2*n* = 12 was found. Interestingly, the two main cytotypes, II and IV, are geographically allopatric (Figure 4A). The cytotypes with fewer chromosome numbers tend to occur at high altitudes, and the relationship between the chromosome number and altitude is significantly negative (*r* = –0.492, *P* < 0.001; Figure 4C).

### Variation in the cpDNA within *Lycoris aurea* complex

The combined sequences of four cpDNA regions were aligned with a consensus length of 4,748 bp. Indels were excluded in the analysis given their proneness to homoplasy. Thus, a total of 4,635 sites excluding sites with gaps/missing data were found in the cpDNA dataset, 76 sites of which were single nucleotide polymorphisms (SNPs) and parsimony-informative (Supplementary Table 3). The region *rpl32-trnL* had the highest proportion of SNPs detected in 881 aligned positions with gaps/missing data excluded (19/881; 2.16%), followed by *ndhF* (17/2,055; 1.8%), *matK* (17/1,180; 1.44%) and *psbA-trnH* (3/519; 0.58%).

**FIGURE 5:**
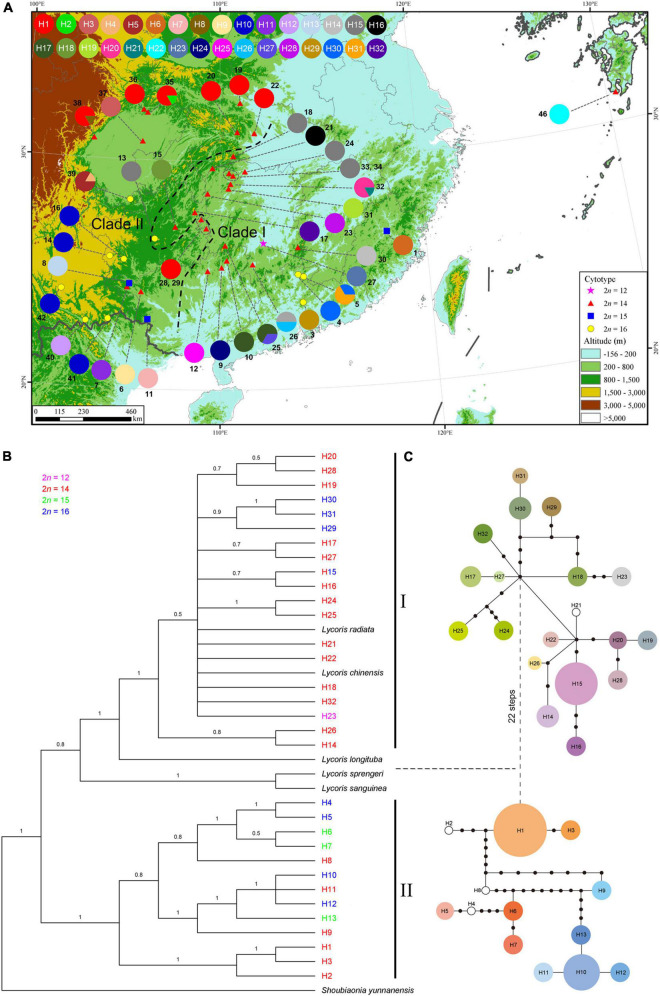
Analysis of cpDNA haplotypes of *L*. *aurea* complex. **(A)** Geographical distribution of the 32 haplotypes across 42 sequenced populations. Pie charts represent haplotype proportions. Colored haplotypes are shared by two or more populations, and blank ones are private haplotypes. Two groups identified by phylogenetic analysis are delimited by black dashed lines. The map was generated by ArcGIS v.9.3 (http://www.esri.com/software/arcgis/arcgis-for-desktop). Elevation data for the map were derived from SRTM elevation data (http://srtm.csi.cgiar.org). Phylogenetic relationships based on the Bayesian analysis **(B)** and network **(C)** for the 32 cpDNA haplotypes detected. The horizontal dashed line between phylogram and network partitions two identified clades (I and II). **(B)** Numbers on the branches represent Bayesian posterior probabilities. Different colors correspond to four cytotypes. **(C)** The sizes of circles in the network are proportional to the frequency of each sampled haplotype, with the smallest circle representing 1 sample and the largest circle representing 45 samples. Black dots represent missing haplotypes (extinct or not found).

These SNPs identified 32 different chloroplast haplotypes (H1-32) across the 42 surveyed populations (Figure 5A and Supplementary Data 1–4). Haplotype frequencies at each locality and geographical distribution are shown in Supplementary Table 4. Of the 32 haplotypes detected, only three haplotypes (H1, H10, and H15) were slightly widespread within different cytotypes. The most common haplotype was H1 (found in 8 of all populations) mainly found in northwestern populations of Hubei and Sichuan with the karyotype of 2*n* = 14 = 8m + 6T (cytotype II), followed by H15 (5/42) and H10 (4/42). Cytotype I having only one population fixed one haplotype (H23). The group of cytotype II, comprising 28 populations surveyed, had the highest number of haplotypes (21/32). Each population within cytotype III fixed a different haplotype, respectively. Cytotype IV with 10 populations harbored 8 haplotypes. Only one haplotype (H15) was shared between cytotypes II and IV.

At the species complex level, the cpDNA data revealed high estimates of haplotype diversity (*H*_d_ = 0.932) and nucleotide diversity (π = 3.96 × 10^–3^). At the cytotype scale, the degrees of cpDNA haplotype diversity and nucleotide diversity were unequal. The cytotype II had the highest haplotype diversity (*H*_d_ = 0.885), with the nucleotide diversity (π) being 3.35 × 10^–3^. The cytotype IV harbored the highest nucleotide diversity (π = 4.09 × 10^–3^), with its haplotype diversity (*H*_d_) being 0.809 (Supplementary Table 4). The AMOVA analyses revealed that 98.61% of the total genetic variation was distributed among populations and 1.39% of the variation within populations (Supplementary Table 5). Within each cytotype, the majority of genetic variation was also distributed among populations (Supplementary Table 5).

The phylogenetic tree of the haplotypes derived from Bayesian inference (Figure 5B) was similar in topology to that from Maximum likelihood analyses (Supplementary Figure 1), and both trees provided strong support for the distinction of two main cpDNA haplotype lineages (Clades I and II), with each lineage including three cytotypes. Both lineages had cytotypes II and IV, however, cytotype I was located in the lineage I and cytotype III was grouped within the lineage II (Figure 5B). Consistent with the phylogenetic trees, the haplotype TCS network also grouped the 32 cpDNA haplotypes into two major clades (Figure 5C), which were connected with each other by 22 mutations. Clade I, comprising 19 haplotypes, occurred in 22 populations of the central and southeastern zones of the distribution range of this complex. There was one exception from the regions, that is, population 2, which was included in clade II. Clade II consisted of 13 haplotypes, which were mainly from the northwestern populations of Hubei and Sichuan and southwestern populations of China.

### Intraspecific variations of leaf morphology within *Lycoris aurea* complex

To identify if morphological characters in different populations or cytotypes of this complex show significant differences, only the populations cultivated in the experimental common garden for more than 4 years to eliminate the influence of environmental factors were selected. The only one population with cytotype I was also excluded. Eventually, 38 populations were analyzed, comprising 25 populations with cytotype II (2*n* = 14), 3 populations with cytotype III (2*n* = 15) and 9 populations with cytotype IV (2*n* = 16).

**FIGURE 6:**
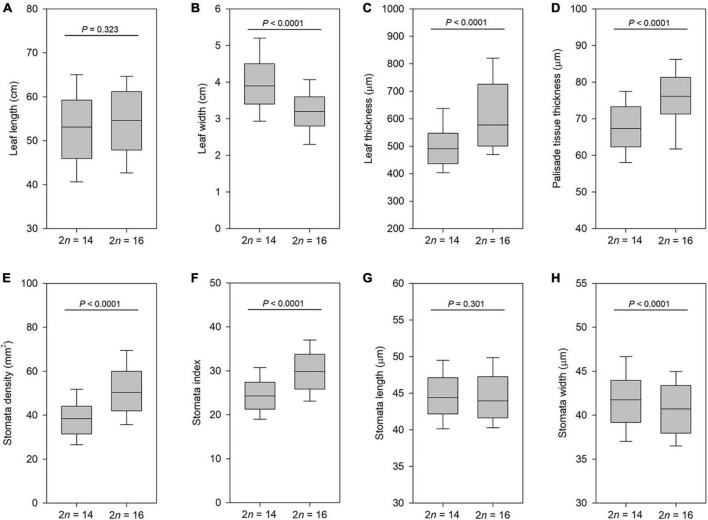
Boxplots of leaf traits in cytotype II (2*n* = 14) and cytotype IV (2*n* = 16). **(A)** Leaf length. **(B)** Leaf width. **(C)** Leaf thickness. **(D)** Palisade tissue thickness. **(E)** Stomata density. **(F)** Stomata index. **(G)** Stomata length. **(H)** Stomata width. Significance was assessed by Student’s *t*-test. *P* < 0.01 means significant at the 0.01 probability level.

We firstly compared the characters of leaf epidermis and size variation of leaf in cytotype II and cytotype IV. For the leaf length no significant differences between cytotype II and cytotype IV were detected (Figure 6A), but the leaf width showed marked differences between them (*P* < 0.001; Figure 6B). The mean leaf width for cytotype II was 4.0 cm, and for cytotype IV was 3.2 cm. Compared to cytotype II, cytotype IV had markedly thicker leaf (*P* < 0.001; Figure 6C) and palisade tissue (*P* < 0.001; Figure 6D). Moreover, stomata density differed between the two cytotypes, and cytotype IV had a higher density of stomata than cytotype II (*P* < 0.001; Figure 6E). The stomata index also revealed significant differences between the two cytotypes (*P* < 0.001; Figure 6F). The stomata length of both cytotypes were nearly identical (Figure 6G), but the stomata width showed significant differences between them (*P* < 0.001; Figure 6H). These results suggested marked morphological differentiation between cytotype II and cytotype IV.

**FIGURE 7:**
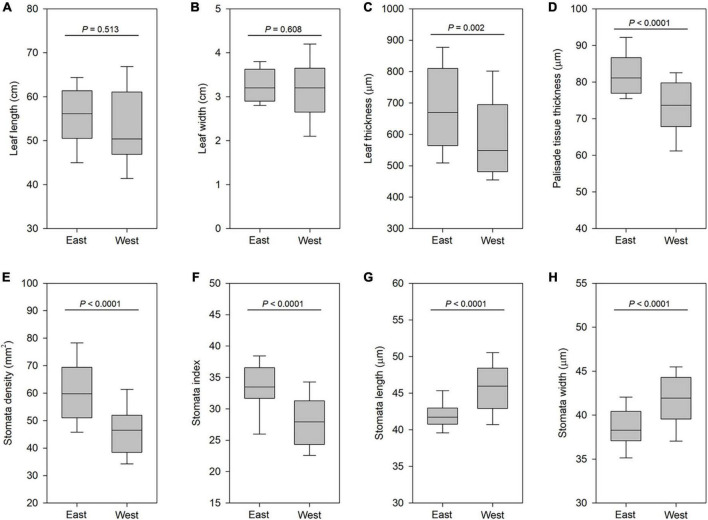
Boxplots of leaf traits in east populations and west populations of cytotype IV (2*n* = 16). **(A)** Leaf length. **(B)** Leaf width. **(C)** Leaf thickness. **(D)** Palisade tissue thickness. **(E)** Stomata density. **(F)** Stomata index. **(G)** Stomata length. **(H)** Stomata width. Significance was assessed by Student’s *t*-test. *P* < 0.01 means significant at the 0.01 probability level.

Next, we addressed whether leaf morphological differentiation among different lineages within the same cytotype, for example, between six southwestern and three southeastern populations with cytotype IV, had occurred. We found no significant differences in both leaf length and leaf width between the six southwestern populations (West) and three southeastern populations (East) (Figures 7A,B). However, the east lineage had significantly thicker leaf (*P* < 0.001; Figure 7C) and palisade tissue (*P* < 0.001; Figure 7D) than the west lineage. Compared with the west lineage, the east lineage had a higher stomata density (*P* < 0.001; Figure 7E). Stomata density for each population varied from 56.3 to 65.7 stomata per mm^2^ on the abaxial epidermis of east lineage and from 37.5 to 55.7 stomata per mm^2^ on the abaxial epidermis of west lineage. The east lineage also had significantly greater stomata index than west lineage (*P* < 0.001; Figure 7F). But west lineage had larger stomata size than east lineage (*P* < 0.001; Figures 7G,H). We found that the apex of ensiform leaves from the west lineage is almost acute, while that in the east lineage is obtuse. These data showed that the divergence of leaf-shaped characters between east and west lineages of cytotype IV had occurred.

We also observed significant differences in leaf characters among three populations with cytotype III (2*n* = 15). For example, the population 11 from Tongde Town has the thinnest leaf (on average 364.2 μm), even among all populations of this complex. Population 1 from Laizhou Town, Fujian has the narrowest leaf among the three populations, with the average leaf width being 2.7 cm, significantly lower than that in population 11 (4.1 cm). We also found that in nature and cultivation, the cytotype III is nearly sterile, and produces no seeds with intact reproductive organs.

## Discussion

### Karyotypic diversity and cytogeography

This study represented the first cytogeographic study of *L*. *aurea* complex. Through detailed karyotypic analyses of *L*. *aurea* complex, considerable karyotypic polymorphism of four distinct cytotypes was found (Table 1). The information provided in the present study, together with previously published chromosome numbers, further unraveled that *L*. *aurea* complex is cytologically complex and that aneuploidy variation has played a very important role in the evolution of the taxa. A previously reported cytotype with 2*n* = 13 = 9m + 4T by Kurita (1987) was not detected in our study, indicating that it was a rare cytotype the same as the cytotype having 2*n* = 12 = 10m + 2T.

Based on an extensive cytogenetical study on *Lycoris* species, Kurita (1987) thought that the genome consisting of five m- and one T-type chromosomes (2*n* = 10m + 2T) was ancestral. In the course of the karyotype evolution, m-type chromosomes changed successively into T-type chromosomes by centromeric fission. Therefore, the genome composed of three m- and five T-type chromosomes (2*n* = 6m + 10T) is a derivative (Kurita, 1987). However, our karyophytogeographical study did not support his view to some extent. The cytotype having 2*n* = 10m + 2T is only found in a restricted small area. Therefore, the widespread cytotypes (2*n* = 8m + 6T or 2*n* = 6m + 10T) may be ancestral.

Previous records (Hsu et al., 1994; Ji and Meerow, 2000; Pei et al., 2017; Quan et al., 2019) showed that *L. aurea* is also distributed in Anhui, southern Jiangsu, southern Shanxi and Zhejiang. However, based on comprehensive sampling and morphological analyses of *Lycoris* species, we found no wild *L. aurea* populations in these regions. For a long time, taxonomists have confused this taxon with another similar yellow-flowered species, *L*. *chinensis*, which is widespread in these regions. But the leaves of *L*. *chinensis* appear in early spring, differing from *L. aurea*.

Cytogeographic patterns may reveal significant amounts of diversity by identifying multiple chromosomal races among related species or within a single taxonomic species and, therefore, contribute to the conservation of rare species and ecological restoration (Soltis et al., 2007; do Pico et al., 2019). The cytogeographic data obtained allowed us to find a clear distribution pattern, since the two most frequent cytotypes, II and IV, occupy different areas and do not co-exist in the same area. The analysis of the geographic distribution of *L. aurea* complex revealed two areas with a high diversity of cytotypes: the junction of Guizhou, Yunnan and Guangxi provinces and the junction of the three provinces, namely Hunan, Jiangxi and Guangdong. It was possible that continuous hybridization and aneuploidization events might have occurred, which would have led to such diversity of cytotypes in these areas.

Compared to the karyotypically-close species *L*. *chinensis* with 2*n* = 16, an extremely low proportion of examined bulbs of *L. aurea* complex has B chromosomes. Our previous work has shown that approximately 10.64% of the total 188 bulbs of *L*. *chinensis* studied have one or more B chromosomes (Liu et al., 2012). Only three B chromosomes were detected in one bulb of the cytotype IV having 2*n* = 16 from population 44. We found no B chromosome in other cytotypes. Whether the B chromosome is specific to the cytotype IV having 2*n* = 16 needs to be tested by checking large sample sizes in the future.

### Multiple origins of sterile cytotype

We found that the cytotype III having 2*n* = 15 = 7m + 8T from these areas was sterile under cultivation. About the origin of this sterile cytotype, there may be three interpretations. The first is that this cytotype was generated from a cytotype having 2*n* = 16 = 6m + 10T by tandem fusion of two among 10 T-type chromosomes. Robertsonian fusion has been found in other *Lycoris* species, *L*. *radiata* (Liu et al., 2019) and *L*. *sanguinea* (Kurita, 1989). The second is that it was evolved from 14-chromosomed cytotype by the fission of one m-type chromosome. The third is that it might be a product of hybridization between the cytotype II having 2*n* = 14 and cytotype IV having 2*n* = 16, due to the fact that the cytotype III was mainly distributed in the regions where cytotype II and cytotype IV met. We speculated that the cytotype III might involve multiple independent origins, corresponding to significantly different leaf-shaped characters in the three populations having 2*n* = 15, rather than a uniform leaf shape from the same clone.

Of the 32 different haplotypes uncovered in *L*. *aurea* complex, 3 occurred in sterile cytotype III. If we assumed that each haplotype evolved only once (without parallel evolution), then we might also conclude that there were a minimum of three independent origins of the sterile cytotypes in the evolutionary history of this complex. If the sterile cytotype was evolved from the fertile cytotypes, II and/or IV, assuming that the haplotype relationship constructed (Figure 5) here was accurate, this pattern implied that some haplotypes in these fertile cytotypes were not detected during our investigation or have gone extinct. Next, we will aim to unravel the accurate origins of the sterile cytotypes by involving multiple nuclear markers and extensive sampling.

### Strong differentiation of *Lycoris aurea* complex

Consistent with extensive karyotypic polymorphism, wide genetic variation was uncovered in the *L*. *aurea* complex. As a member of the Sino-Japanese flora, this complex had extremely high genetic diversity. At the species level, the haplotype diversity (*H*_d_ = 0.932) revealed by cpDNA data was higher than the mean value of 26 species in the Sino-Japanese Floristic Region (SJFR) of East Asia summarized and reported by Zhang et al. (2022) recently. For example, at the genus-wide scale, *Cardiocrinum* (Endlicher) Lindley, a genus containing three species and one variety of bulbous perennial herbs in the SJFR (Yang et al., 2017), has a markedly lower cpDNA haplotype diversity (*H*_d_ = 0.792) than that at the *L*. *aurea* species-level.

AMOVA analysis showed that the majority of genetic variation (98.61%) in cpDNA was attributed to inter-population variability, indicating that this complex has undergone strong population differentiation and very limited gene flow. Our cpDNA phylogenetic trees demonstrated that *L*. *aurea* complex was not monophyletic and differentiated into two distinct major lineages. Three other *Lycoris* species, i.e., *L. radiata*, *L. chinensis*, and *L. longituba*, were grouped within lineage I with high support (PP = 1, BS = 97%), and another two *Lycoris* species, *L. sprengeri* and *L. sanguinea*, were also close to lineage I, despite relatively low support (PP = 0.8, BS = 55%). When cultivated in the common environment for a long time, significant leaf character differences between and within cytotypes were found, suggesting that high variation in leaf morphological characters within the *L*. *aurea* complex is mainly the product of genetic variation. Moreover, different cytotypes and/or different lineages with the same chromosome number had non-overlapping distribution ranges. These results implied that *L*. *aurea* complex had experienced marked bio-geographical diversification and might contain multiple species. The taxonomic and evolutionary problems, which should be addressed in future, are the accurate species delimitation through an integrative taxonomic approach and the trend of karyotypic evolution by combining phylogenomics and fluorescence *in situ* hybridization analyses.

## Conclusion

Our cytogeographic and plastid DNA haplotype analysis indicated that *L*. *aurea* comprised more than two monophyletic lineages with a deep divergence. Moreover, several morphological diagnostic characteristics were discovered from the field and common garden that corresponded to these independent evolutionary lineages. For example, the fertile cytotype (2*n* = 16) contained two main independent lineages, which are also geographically allopatric. The populations having 2n = 16 from Guangdong province have obtuse leaf apex, whereas the leaf apex of all other *L*. *aurea* populations is acute, suggesting the former was a distinct species. Taking into account all of this evidence, we considered *L*. *aurea* complex should comprise multiple independent evolutionary lineages. Accurate species delimitation needs to be established, both for assisting wild resource conservation and reasonable utilization. There has been more long-standing interest in polyploidy than aneuploidy in plants. The importance of polyploidization in plant evolution has been highlighted. Future studies should focus on trying to understand the role of aneuploidization in flowering plant evolution and speciation.

## Data availability statement

The data presented in this study are deposited in the GenBank repository, accession number: OP021502 – OP021631.

## Author contributions

KL conceived and designed the article. KL, WM, and DZ contributed to the sampling. JW, LS, HZ, YL, and GL performed the experiment. JW, LS, and KL analyzed the data and wrote the manuscript. All authors contributed to the article and approved the submitted version.

## Footnotes

1
www.sangon.com
