# Supplementary material for: Aneuploidy promotes intraspecific diversification of the endemic East Asian herb Lycoris aurea complex
Source: Front Plant Sci. 2022 Sep 29;13:955724. doi: 10.3389/fpls.2022.955724 (PMC9558133; doi:10.3389/fpls.2022.955724)
Supplement: Supplementary file 1 [file Data_Sheet_5.docx]

**Supplementary material**

The following Supplementary material is available for this article:

**Figure S1.** Phylogenetic tree based on the Maximum likelihood analyses.

**Table S1.** Primers for amplification and sequencing of cpDNA regions.

**Table S2.** Measurements of somatic chromosomes in four representative cytotypes.

**Table S3.** Variable SNP sites of cpDNA sequence in 32 haplotypes of *L. aurea* complex.

**Table S4.** Genetic diversity and cpDNA haplotypes of *L*. *aurea* complex.

**Table S5.** Results of analysis of molecular variance (AMOVA) for all populations and cytotypes of *L*. *aurea* complex.

**Data S1.** The multiple alignment of *matK* sequences of thirty-two haplotypes.

**Data S2.** The multiple alignment of *rpl32-trnL* sequences of thirty-two haplotypes.

**Data S3.** The multiple alignment of *psbA-trnH* sequences of thirty-two haplotypes.

**Data S4.** The multiple alignment of *ndhF* sequences of thirty-two haplotypes.


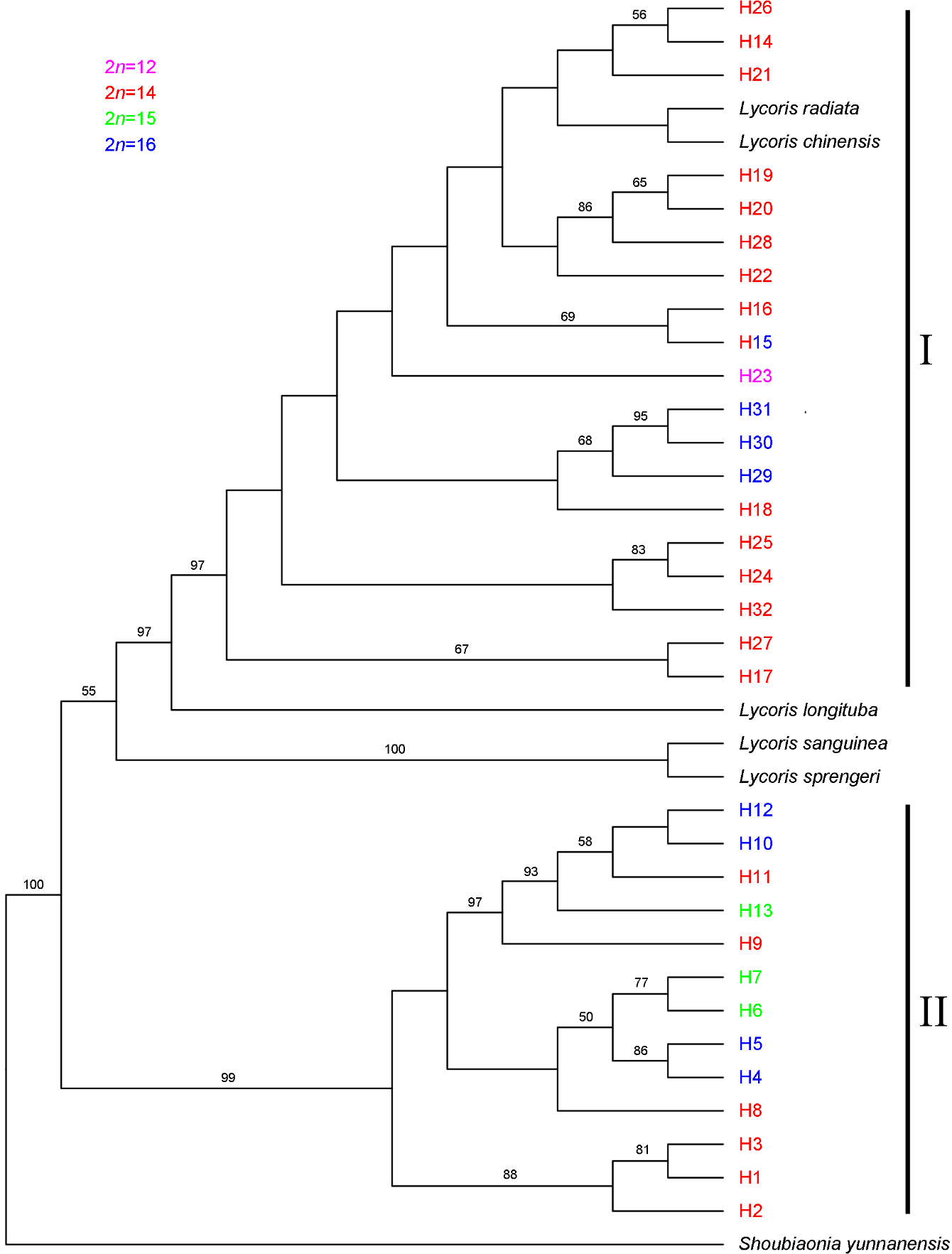


Figure S1. Phylogenetic tree based on the Maximum likelihood analyses. Numbers on the branches represent bootstrap support.

Table S1. Primers for amplification and sequencing of cpDNA regions

| **Primer Name** | **Sequence (5' to 3')** |
| --- | --- |
| *matK*-1F | AAAGTTCTAGCACACGAAAGTCGA |
| *matK*-2F | CAAAGAAAAAGAAATACCAAA |
| *matK*-R | CTATATCCGCTACTCTTTCAGGAGT |
| *rpl32*-F | CAGTTCCAAAAAAACGTACTTCTA |
| *trnL*-R | CTGCTTCCTAAGAGCAGCGTGTCT |
| *psbA*-F | CGCGCGTGGTGGATTCACAATCC |
| *trnH*-R | GTAATGCACGAACGTAATGCTC |
| *ndhF*-1F | ATGGAACATACATATAAATATGCATGG |
| *ndhF*-2F | TTTTGTAACCAATCGTATAGG |
| *ndhF*-3F | GCCTATTCGAAAGCTTTATTGT |
| *ndhF*-4F | GAGTACGATACTTTAGTACTT |
| *ndhF*-R | CCCCATGTATTTGATCCCCTCTCC |

Table S2. Measurements of somatic chromosomes in four representative cytotypes

| Cytotypes | Chromosome | Relative length | | | Arm ratio | Type |
| --- | --- | --- | --- | --- | --- | --- |
|  |  | SL | LL | TL |  |  |
|  | Pair 1 | 10.9 | 10.05 | 20.95 | 1.08 | m |
|  | Pair 2 | 10.21 | 9.34 | 19.54 | 1.09 | m |
| Cytotype Ⅰ | Pair 3 | 9.83 | 8.56 | 18.39 | 1.15 | m |
|  | Pair 4 | 8.73 | 7.84 | 16.57 | 1.11 | m |
|  | Pair 5 | 8.22 | 7.22 | 15.44 | 1.14 | m |
|  | Pair 6 | 9.11 | 0 | 9.11 | ∞ | T |
|  | Pair 1 | 10.32 | 9.46 | 19.78 | 1.09 | m |
|  | Pair 2 | 9.68 | 8.92 | 18.6 | 1.08 | m |
| Cytotype Ⅱ | Pair 3 | 9.4 | 8.53 | 17.93 | 1.1 | m |
|  | Pair 4 | 8.62 | 7.91 | 16.53 | 1.09 | m |
|  | Pair 5 | 9.44 | 0 | 9.44 | ∞ | T |
|  | Pair 6 | 9.09 | 0 | 9.09 | ∞ | T |
|  | Pair 7 | 8.64 | 0 | 8.64 | ∞ | T |
|  | No. 1 | 5.42 | 5.21 | 10.63 | 1.04 | m |
|  | No. 2 | 4.96 | 4.69 | 9.65 | 1.06 | m |
|  | No. 3 | 4.88 | 4.7 | 9.58 | 1.04 | m |
|  | No. 4 | 4.85 | 4.52 | 9.36 | 1.07 | m |
|  | No. 5 | 4.7 | 4.42 | 9.12 | 1.06 | m |
|  | No. 6 | 4.68 | 4.24 | 8.92 | 1.1 | m |
| Cytotype Ⅲ | No. 7 | 4.46 | 3.79 | 8.25 | 1.18 | m |
|  | No. 8 | 4.92 | 0 | 4.92 | ∞ | T |
|  | No. 9 | 4.78 | 0 | 4.78 | ∞ | T |
|  | No. 10 | 4.56 | 0 | 4.56 | ∞ | T |
|  | No. 11 | 4.4 | 0 | 4.4 | ∞ | T |
|  | No. 12 | 4.2 | 0 | 4.2 | ∞ | T |
|  | No. 13 | 3.93 | 0 | 3.93 | ∞ | T |
|  | No. 14 | 3.9 | 0 | 3.9 | ∞ | T |
|  | No. 15 | 3.8 | 0 | 3.8 | ∞ | T |
|  | Pair 1 | 10.74 | 9.46 | 20.2 | 1.13 | m |
|  | Pair 2 | 10.08 | 8.9 | 18.98 | 1.13 | m |
|  | Pair 3 | 9.19 | 8.74 | 17.93 | 1.05 | m |
| Cytotype Ⅳ | Pair 4 | 10.15 | 0 | 10.15 | ∞ | T |
|  | Pair 5 | 8.99 | 0 | 8.99 | ∞ | T |
|  | Pair 6 | 8.33 | 0 | 8.33 | ∞ | T |
|  | Pair 7 | 7.94 | 0 | 7.94 | ∞ | T |
|  | Pair 8 | 7.49 | 0 | 7.49 | ∞ | T |

Note: SL, relative length of short arm; LL, relative length of long arm; TL, total relative length; SL + LL = TL. Cytotypes Ⅰ, Ⅱ, Ⅲ and Ⅳ are from populations 27, 23, 11 and 4, respectively.

|  | | | | | | | | | | | | | | | | | | | | | | | | | | | | | | | | | | | |  |
| --- | --- | --- | --- | --- | --- | --- | --- | --- | --- | --- | --- | --- | --- | --- | --- | --- | --- | --- | --- | --- | --- | --- | --- | --- | --- | --- | --- | --- | --- | --- | --- | --- | --- | --- | --- | --- |
| Table S3. Variable SNP sites of cpDNA sequence in 32 haplotypes of *L. aurea* complex | | | | | | | | | | | | | | | | | | | | | | | | | | | | | | | | | | | |  |
|  | **Variable SNP Sites** | | | | | | | | | | | | | | | | | | | | | | | | | | | | | | | | | | |  |
| **Haplotype** | ***matK*** | | | | | | | | | | | | | | | | | ***rpl*32-*trn*L** | | | | | | | | | | | | | | | | | |  |
|  | 7 3 | 1 2 8 | 3 2 6 | 3 6 5 | 4 3 1 | 4 9 0 | 5 3 1 | 6 0 5 | 6 6 7 | 6 7 0 | 7 3 5 | 8 4 9 | 8 8 6 | 9 1 4 | 9 3 0 | 1 0 7 8 | 1 1 0 4 | 1 3 1 7 | 1 3 2 5 | 1 3 7 4 | 1 3 8 5 | 1 4 1 8 | 1 4 2 3 | 1 4 5 1 | 1 4 9 3 | 1 5 8 8 | 1 6 1 7 | 1 6 4 7 | 1 6 8 7 | 1 6 9 9 | 1 7 5 2 | 1 7 5 8 | 1 7 7 6 | 1 9 4 4 | 2 0 4 4 | 2 0 5 6 |
| H1 | C | T | G | C | A | A | G | A | T | A | C | T | A | G | C | A | G | T | A | A | A | T | T | T | G | A | A | G | A | A | A | C | C | T | C | A |
| H2 | • | • | • | • | T | • | • | • | • | • | • | C | • | T | T | • | • | • | • | • | • | • | • | • | • | • | • | • | • | • | • | • | • | • | • | • |
| H3 | • | • | • | • | • | • | • | • | • | • | • | • | • | • | • | • | • | • | • | • | • | • | • | • | • | • | • | • | • | • | • | • | • | • | • | • |
| H4 | • | C | • | • | • | • | • | • | • | • | • | C | • | • | • | • | • | • | • | • | G | • | • | C | • | • | • | • | • | • | • | • | • | • | • | • |
| H5 | • | C | • | • | • | • | • | • | • | • | • | C | • | • | • | • | • | • | • | • | • | • | • | • | • | • | • | • | • | • | • | • | • | • | • | • |
| H6 | • | C | • | • | • | • | • | • | • | • | • | C | • | • | • | • | • | • | • | • | G | • | • | C | • | • | • | • | • | T | • | T | • | • | • | • |
| H7 | • | C | • | • | • | • | • | • | • | • | • | C | • | • | • | • | • | • | • | • | G | • | • | C | • | • | • | • | • | T | • | T | • | • | • | • |
| H8 | • | C | • | • | • | • | • | • | • | • | • | C | • | • | • | • | • | • | • | • | G | • | • | C | • | • | • | • | • | T | • | T | • | • | • | • |
| H9 | • | • | • | • | T | G | • | • | • | • | • | C | • | T | T | • | • | • | • | T | G | • | • | C | • | • | • | • | • | T | • | T | • | • | • | • |
| H10 | • | • | • | • | T | G | • | • | • | • | • | C | • | T | T | • | • | • | • | T | G | • | • | C | • | • | • | • | • | T | • | T | • | • | • | • |
| H11 | • | • | • | • | T | G | • | • | • | • | • | C | • | T | T | • | • | • | • | T | G | • | • | C | • | • | • | • | • | T | • | T | • | • | • | • |
| H12 | • | • | • | • | T | G | • | • | • | • | • | C | • | T | T | • | • | • | • | T | G | • | • | C | • | • | • | • | • | T | • | T | • | • | • | • |
| H13 | • | • | • | • | T | G | • | • | • | • | • | C | • | T | T | • | • | • | • | T | G | • | • | C | • | • | • | • | • | T | • | T | • | • | • | • |
| H14 | T | • | • | T | • | • | C | G | • | • | • | • | • | • | • | G | • | C | T | • | G | C | • | C | T | C | C | • | • | • | C | • | A | • | • | G |
| H15 | T | • | • | T | • | • | C | G | • | • | • | • | • | • | • | G | • | C | T | • | G | C | • | C | • | • | • | • | • | • | C | • | A | • | • | • |
| H16 | T | • | • | T | • | • | C | G | • | • | • | • | • | • | • | G | • | C | T | • | G | C | • | C | • | • | • | • | • | • | C | • | A | • | • | • |
| H17 | T | • | • | T | • | • | C | G | • | • | • | • | • | • | • | G | • | C | T | • | G | C | • | C | • | • | • | • | T | • | C | • | A | • | • | • |
| H18 | T | • | • | T | • | • | C | G | • | • | • | • | • | • | • | G | • | C | T | • | G | C | • | C | • | • | • | • | • | • | C | • | A | • | • | • |

|  |  |  |  |  |  |  |  |  |  |  |  |  |  |  |  |  |  |  |  |  |  |  |  |  |  |  |  |  |  |  |  |  |  |  |  |  |
| --- | --- | --- | --- | --- | --- | --- | --- | --- | --- | --- | --- | --- | --- | --- | --- | --- | --- | --- | --- | --- | --- | --- | --- | --- | --- | --- | --- | --- | --- | --- | --- | --- | --- | --- | --- | --- |
|  | **Variable SNP Sites** | | | | | | | | | | | | | | | | | | | | | | | | | | | | | | | | | | |  |
| **Haplotype** | ***matK*** | | | | | | | | | | | | | | | | | ***rpl*32-*trn*L** | | | | | | | | | | | | | | | | | |  |
|  | 7 3 | 1 2 8 | 3 2 6 | 3 6 5 | 4 3 1 | 4 9 0 | 5 3 1 | 6 0 5 | 6 6 7 | 6 7 0 | 7 3 5 | 8 4 9 | 8 8 6 | 9 1 4 | 9 3 0 | 1 0 7 8 | 1 1 0 4 | 1 3 1 7 | 1 3 2 5 | 1 3 7 4 | 1 3 8 5 | 1 4 1 8 | 1 4 2 3 | 1 4 5 1 | 1 4 9 3 | 1 5 8 8 | 1 6 1 7 | 1 6 4 7 | 1 6 8 7 | 1 6 9 9 | 1 7 5 2 | 1 7 5 8 | 1 7 7 6 | 1 9 4 4 | 2 0 4 4 | 2 0 5 6 |
| H19 | T | • | A | T | • | • | C | G | • | • | G | • | • | • | • | G | • | C | T | • | G | C | • | C | • | • | • | • | • | • | C | • | A | • | T | • |
| H20 | T | • | A | T | • | • | C | G | • | • | • | • | • | • | • | G | • | C | T | • | G | C | • | C | • | • | • | • | • | • | C | • | A | • | T | • |
| H21 | T | • | • | T | • | • | C | G | C | • | • | • | • | • | • | G | • | C | T | • | G | C | • | C | • | • | • | • | • | • | C | • | A | • | • | • |
| H22 | T | • | • | T | • | • | C | G | • | • | • | • | • | • | • | G | A | C | T | • | G | C | • | C | • | • | • | • | • | • | C | • | A | • | • | • |
| H23 | T | • | • | T | • | • | C | G | • | • | • | • | • | • | • | G | • | C | T | • | G | C | C | C | • | • | • | • | • | • | C | • | A | • | • | • |
| H24 | T | • | • | T | • | • | C | G | • | • | • | G | T | • | • | G | • | C | T | • | G | C | • | C | • | • | • | • | • | • | C | • | A | • | • | • |
| H25 | T | • | • | T | • | • | C | G | • | • | • | • | T | • | • | G | • | C | T | • | G | C | • | C | • | • | • | • | • | • | C | • | A | • | • | • |
| H26 | T | • | • | T | • | • | C | G | • | • | • | • | • | • | • | G | • | C | T | • | G | C | • | C | • | C | • | • | • | • | C | • | A | C | • | • |
| H27 | T | • | • | T | • | • | C | G | • | • | • | • | • | • | • | G | • | C | T | • | G | C | • | C | • | • | • | • | • | • | C | • | A | • | • | • |
| H28 | T | • | A | T | • | • | C | G | • | • | • | • | • | • | • | G | • | C | T | • | G | C | • | C | • | • | • | • | • | • | C | • | A | • | T | • |
| H29 | T | • | • | T | • | • | C | G | • | G | • | G | • | • | • | G | • | C | T | • | G | C | • | C | • | • | • | • | • | • | C | • | A | • | • | • |
| H30 | T | • | • | T | • | • | C | G | • | G | • | • | • | • | • | G | • | C | T | • | G | C | • | C | • | • | • | • | • | • | C | • | A | • | • | • |
| H31 | T | • | • | T | • | • | C | G | • | G | • | • | • | • | • | G | • | C | T | • | G | C | • | C | • | • | • | A | • | • | C | • | A | • | • | • |
| H32 | T | • | • | T | • | • | C | G | • | • | • | • | • | • | • | G | • | C | T | • | G | C | • | C | • | • | • | • | • | • | C | • | A | • | • | • |

|  |  |  |  |  |  |  |  |  |  |  |  |  |  |  |  |  |  |  |  |  |  |  |  |  |  |  |  |  |  |  |  |  |  |  |  |  |  |  |  |  |
| --- | --- | --- | --- | --- | --- | --- | --- | --- | --- | --- | --- | --- | --- | --- | --- | --- | --- | --- | --- | --- | --- | --- | --- | --- | --- | --- | --- | --- | --- | --- | --- | --- | --- | --- | --- | --- | --- | --- | --- | --- |
|  |  | | | | | | | | | | | | | | | | | | | | | | | | | | | | | | | | | | | | | | | |
| **Haplotype** | ***psb*A-*trn*H** | | | ***ndhF*** | | | | | | | | | | | | | | | | | | | | | | | | | | | | | | | | | | | | |
|  | 2 6 2 2 | 2 6 2 6 | 2 6 5 2 | 2 7 5 7 | 2 7 5 8 | 2 7 9 7 | 2 8 6 0 | 3 0 8 1 | 3 2 3 9 | 3 3 1 8 | 3 3 1 9 | 3 4 1 3 | 3 5 0 3 | 3 5 5 4 | 3 7 1 0 | 3 9 4 7 | 3 9 5 5 | 4 0 0 6 | 4 0 1 6 | 4 0 1 7 | 4 0 1 8 | 4 0 6 4 | 4 0 7 7 | 4 1 7 4 | 4 1 7 9 | 4 2 4 1 | 4 2 5 9 | 4 2 6 1 | 4 3 1 1 | 4 3 7 0 | 4 3 7 1 | 4 3 9 1 | 4 4 2 1 | 4 4 2 9 | 4 4 3 8 | 4 4 3 9 | 4 5 7 1 | 4 6 3 7 | 4 6 4 2 | 4 7 0 9 |
| H1 | C | C | A | C | C | C | T | T | A | G | C | A | G | A | C | A | C | G | T | C | A | A | G | G | A | G | T | T | A | G | G | G | G | C | C | G | C | T | T | C |
| H2 | • | • | • | • | • | • | • | • | • | • | • | • | • | • | • | • | • | • | • | • | • | • | • | • | • | • | • | • | • | • | • | • | • | • | • | • | • | • | • | • |
| H3 | • | • | • | T | • | • | • | • | • | • | • | • | • | • | • | • | • | A | • | • | • | • | • | • | • | • | • | • | • | • | • | • | • | • | • | • | • | • | • | • |
| H4 | • | • | T | • | • | T | • | • | • | • | • | G | • | G | • | • | • | • | • | • | • | • | • | • | G | • | • | • | • | A | • | • | • | • | • | • | • | G | • | • |
| H5 | • | • | T | • | • | T | • | • | • | • | • | G | • | • | • | • | • | • | • | • | • | • | • | • | G | • | • | • | • | A | • | • | • | • | • | • | • | G | • | • |
| H6 | • | • | T | • | • | T | • | • | • | • | • | G | • | • | • | • | • | • | • | • | • | • | • | • | • | • | • | • | • | • | • | • | • | • | • | • | • | G | • | • |
| H7 | • | • | T | • | • | T | • | G | • | • | • | G | • | • | • | • | • | • | • | • | • | • | • | • | • | • | • | • | • | • | T | • | • | • | • | • | • | G | • | • |
| H8 | • | • | T | • | • | • | • | • | • | • | • | • | • | • | • | • | • | • | • | • | • | • | • | • | • | • | • | • | • | • | • | • | • | • | • | • | • | • | • | • |
| H9 | A | • | T | • | • | • | • | • | • | • | • | G | • | G | • | • | • | • | • | • | • | • | • | • | • | • | • | • | • | • | • | • | • | • | • | • | • | • | • | • |
| H10 | A | • | T | • | • | • | • | G | • | • | • | G | • | G | • | G | • | • | • | • | • | G | A | • | • | • | • | • | • | • | • | • | A | • | • | • | • | G | • | • |
| H11 | A | • | T | • | • | • | • | G | • | • | • | G | • | G | • | G | • | • | • | • | • | G | A | • | • | • | • | • | • | • | • | • | A | • | T | • | • | G | • | • |
| H12 | C | • | T | • | • | • | • | G | • | • | • | G | • | G | • | G | • | • | • | • | • | G | A | • | • | • | • | • | • | • | • | • | A | • | • | • | • | G | • | • |
| H13 | A | • | T | • | • | • | • | • | • | • | • | G | • | G | • | G | • | • | • | • | • | G | A | • | • | • | • | • | • | • | • | • | A | • | • | • | • | G | • | • |
| H14 | A | T | T | • | • | • | • | • | • | A | T | G | • | G | • | • | • | • | • | A | • | • | • | • | • | A | • | C | • | • | • | • | • | • | • | • | • | • | • | • |
| H15 | A | T | T | • | A | • | • | • | • | A | T | G | • | G | • | • | • | • | • | A | • | • | • | • | • | A | • | C | • | • | • | T | • | • | • | • | • | • | • | • |
| H16 | A | T | T | • | A | • | • | G | • | A | T | G | • | G | • | • | • | • | A | A | • | • | • | • | • | A | • | C | • | • | • | T | • | • | • | A | • | • | • | • |
| H17 | A | T | T | • | • | • | • | • | • | A | T | G | • | G | • | • | • | • | • | • | • | • | • | • | • | A | • | C | • | • | • | • | • | • | • | • | • | • | C | • |
| H18 | A | T | T | • | • | • | • | G | • | A | T | G | • | G | • | • | • | • | • | • | • | • | • | • | • | A | • | C | • | • | • | • | • | • | • | • | • | • | • | • |

|  |  |  |  |  |  |  |  |  |  |  |  |  |  |  |  |  |  |  |  |  |  |  |  |  |  |  |  |  |  |  |  |  |  |  |  |  |  |  |  |  |
| --- | --- | --- | --- | --- | --- | --- | --- | --- | --- | --- | --- | --- | --- | --- | --- | --- | --- | --- | --- | --- | --- | --- | --- | --- | --- | --- | --- | --- | --- | --- | --- | --- | --- | --- | --- | --- | --- | --- | --- | --- |
|  |  | | | | | | | | | | | | | | | | | | | | | | | | | | | | | | | | | | | | | | | |
| **Haplotype** | ***psb*A-*trn*H** | | | ***ndhF*** | | | | | | | | | | | | | | | | | | | | | | | | | | | | | | | | | | | | |
|  | 2 6 2 2 | 2 6 2 6 | 2 6 5 2 | 2 7 5 7 | 2 7 5 8 | 2 7 9 7 | 2 8 6 0 | 3 0 8 1 | 3 2 3 9 | 3 3 1 8 | 3 3 1 9 | 3 4 1 3 | 3 5 0 3 | 3 5 5 4 | 3 7 1 0 | 3 9 4 7 | 3 9 5 5 | 4 0 0 6 | 4 0 1 6 | 4 0 1 7 | 4 0 1 8 | 4 0 6 4 | 4 0 7 7 | 4 1 7 4 | 4 1 7 9 | 4 2 4 1 | 4 2 5 9 | 4 2 6 1 | 4 3 1 1 | 4 3 7 0 | 4 3 7 1 | 4 3 9 1 | 4 4 2 1 | 4 4 2 9 | 4 4 3 8 | 4 4 3 9 | 4 5 7 1 | 4 6 3 7 | 4 6 4 2 | 4 7 0 9 |
| H19 | A | T | T | • | • | • | • | • | • | A | T | G | • | G | • | • | • | • | • | A | • | • | • | • | • | A | • | C | • | • | • | • | • | • | • | • | • | • | • | • |
| H20 | A | T | T | • | • | • | • | • | • | A | T | G | • | G | • | • | • | • | • | A | • | • | • | • | • | A | • | C | • | • | • | • | • | • | • | • | • | • | • | • |
| H21 | A | T | T | • | • | • | • | • | • | A | T | G | • | G | • | • | • | • | • | A | • | • | • | • | • | A | • | C | • | • | • | • | • | • | • | • | • | • | • | • |
| H22 | A | T | T | • | • | • | • | • | • | A | T | G | • | G | • | • | • | • | • | A | • | • | • | • | • | A | • | C | • | • | • | • | • | • | • | • | • | • | • | • |
| H23 | A | T | T | • | • | • | • | G | • | A | T | G | • | G | • | • | • | • | • | A | • | • | • | • | • | A | • | C | C | • | • | • | • | • | • | • | • | • | • | • |
| H24 | A | T | T | • | • | • | • | • | • | A | T | G | • | G | T | • | • | • | • | • | • | • | • | A | • | A | • | C | • | • | • | • | • | A | • | • | • | • | • | • |
| H25 | A | T | T | • | • | • | • | • | C | A | T | G | T | G | • | • | • | • | • | • | • | • | • | • | • | A | • | C | • | • | • | • | • | A | • | • | • | • | • | • |
| H26 | A | T | T | • | • | • | • | • | • | A | T | G | • | G | • | • | • | • | • | A | • | • | • | • | • | A | • | C | • | • | • | • | • | • | • | • | • | • | • | • |
| H27 | A | T | T | • | • | • | • | • | • | A | T | G | • | G | • | • | • | • | • | • | • | • | • | • | • | A | • | C | • | • | • | • | • | • | • | • | • | • | C | • |
| H28 | A | T | T | • | • | • | • | • | • | A | T | G | • | G | • | • | • | • | • | T | T | • | • | • | • | A | • | C | • | • | • | • | • | • | • | • | • | • | • | • |
| H29 | A | T | T | • | • | • | • | G | • | A | T | G | • | G | • | • | T | A | • | • | • | • | • | • | • | A | C | C | • | • | • | • | • | • | • | • | • | • | • | • |
| H30 | A | T | T | • | • | • | • | • | • | A | T | G | • | G | • | • | • | A | • | • | • | • | • | • | • | A | C | C | • | • | • | • | • | • | • | • | A | • | • | • |
| H31 | A | T | T | • | • | • | • | • | • | A | T | G | • | G | • | • | • | A | • | • | • | • | • | • | • | A | C | C | • | • | • | • | • | • | • | • | A | • | • | • |
| H32 | A | T | T | • | • | • | G | • | • | A | T | G | • | G | • | • | • | • | • | • | • | • | • | • | • | A | • | C | • | • | • | • | • | • | • | • | • | • | • | T |
|  | Notes: •, same with H1 loci. | | | | | | | | | | | | | | | | | | | | | | | | | | | | | | | | | | | | | | | |

|  |
| --- |

Table S4. Genetic diversity and cpDNA haplotypes of *L*. *aurea* complex

| Karyotypes | Populations | Individuals | Haplotype diversity (*H*_d_) | Nucleotide diversity (π×10^-3^) | Haplotypes (H) |
| --- | --- | --- | --- | --- | --- |
| 2*n* = 12 | Pop27 | 6 | 0 | 0 | H23(6) |
|  | Cytotype Ⅰ |  | 0 | 0 |  |
| 2*n* = 14 | Pop6 | 6 | 0 | 0 | H9(6) |
|  | Pop7 | 6 | 0 | 0 | H11(6) |
|  | Pop9 | 6 | 0 | 0 | H24(6) |
|  | Pop10 | 3 | 0 | 0 | H17(3) |
|  | Pop12 | 6 | 0 | 0 | H25(6) |
|  | Pop15 | 6 | 0 | 0 | H18(6) |
|  | Pop17 | 6 | 0 | 0 | H32(6) |
|  | Pop18 | 6 | 0 | 0 | H15(6) |
|  | Pop19 | 5 | 0 | 0 | H1(5) |
|  | Pop20 | 6 | 0 | 0 | H1(6) |
|  | Pop21 | 6 | 0 | 0 | H16(6) |
|  | Pop22 | 6 | 0 | 0 | H1(6) |
|  | Pop23 | 6 | 0 | 0 | H28(6) |
|  | Pop24 | 6 | 0 | 0 | H15(6) |
|  | Pop25 | 6 | 0.533 | 0.12 | H17(4), H27(2) |
|  | Pop26 | 6 | 0.6 | 0.39 | H14(3), H26(3) |
|  | Pop28 | 6 | 0 | 0 | H1(6) |
|  | Pop29 | 6 | 0 | 0 | H1(6) |
|  | Pop30 | 5 | 0 | 0 | H14(5) |
|  | Pop31 | 6 | 0 | 0 | H19(6) |
|  | Pop32 | 6 | 0.333 | 0.22 | H20(5), H21(1) |
|  | Pop33 | 5 | 0 | 0 | H15(5) |
|  | Pop34 | 6 | 0 | 0 | H15(6) |
|  | Pop35 | 6 | 0.333 | 0.29 | H1(5), H2(1) |
|  | Pop36 | 6 | 0 | 0 | H1(6) |
|  | Pop37 | 6 | 0 | 0 | H3(6) |
|  | Pop38 | 6 | 0.333 | 0.5 | H1(5), H8(1) |
|  | Pop46 | 4 | 0 | 0 | H22(4) |
|  | Cytotype Ⅱ |  | 0.885 | 3.35 |  |
| 2*n* = 15 | Pop1 | 6 | 0 | 0 | H6(6) |
|  | Pop8 | 6 | 0 | 0 | H13(6) |
|  | Pop11 | 6 | 0 | 0 | H7(6) |
|  | Cytotype Ⅲ |  | 0.706 | 1.42 |  |
| 2*n* = 16 | Pop3 | 6 | 0 | 0 | H29(6) |
|  | Pop4 | 6 | 0 | 0 | H30(6) |
|  | Pop5 | 6 | 0.533 | 0.12 | H30(2), H31(4) |
|  | Pop13 | 6 | 0 | 0 | H15(6) |
|  | Pop14 | 3 | 0 | 0 | H10(3) |
|  | Pop16 | 6 | 0 | 0 | H10(6) |
|  | Pop39 | 6 | 0.333 | 0.14 | H4(1), H5(5) |
|  | Pop40 | 6 | 0 | 0 | H12(6) |
|  | Pop41 | 6 | 0 | 0 | H10(6) |
|  | Pop42 | 6 | 0 | 0 | H10(6) |
|  | Cytotype Ⅳ |  | 0.809 | 4.09 |  |
|  | Total |  | 0.932 | 3.96 |  |

Table S5. Results of analysis of molecular variance (AMOVA) for all populations and cytotypes of *L*. *aurea* complex

| Source of variation | *d.f.* | SS | VC | PV | Fixation index |
| --- | --- | --- | --- | --- | --- |
| All populations |  |  |  |  | *F*_ST_ = 0.986** |
| Among populations | 41 | 7993.082 | 33.905 | 98.61 |  |
| Within populations | 199 | 95.200 | 0.478 | 1.39 |  |
| Total | 240 | 8088.282 | 34.384 |  |  |
| Cytotype Ⅱ |  |  |  |  | *F*_ST_ = 0.983** |
| Among populations | 27 | 1221.112 | 7.896 | 98.35 |  |
| Within populations | 132 | 17.500 | 0.133 | 1.65 |  |
| Total | 159 | 1238.612 | 8.028 |  |  |
| Cytotype Ⅲ |  |  |  |  | *F*_ST_ = 1** |
| Among populations | 2 | 56.000 | 4.667 | 100 |  |
| Within populations | 15 | 0.000 | 0.000 | 0.00 |  |
| Total | 17 | 56.000 | 4.667 |  |  |
| Cytotypes Ⅳ |  |  |  |  | *F*_ST_ = 0.994** |
| Among populations | 9 | 530.895 | 10.366 | 99.39 |  |
| Within populations | 47 | 3.000 | 0.064 | 0.61 |  |
| Total | 56 | 533.895 | 10.430 |  |  |

*d.f.*, degrees of freedom; *F*_ST_, correlation within populations relative to total; PV, percentage of variation; SS, sum of squares; VC, variance component; ***P*<0.01, 1000 permutations
